# Supplementary material for: Magnitude of asymptomatic COVID-19 cases throughout the course of infection: A systematic review and meta-analysis
Source: PLoS One. 2021 Mar 23;16(3):e0249090. doi: 10.1371/journal.pone.0249090 (PMC7987199; doi:10.1371/journal.pone.0249090)

**S1 Table.** Individual effect sizes of the included studies conducted on truly asymptomatic SARS-CoV-2 infection, 2020.


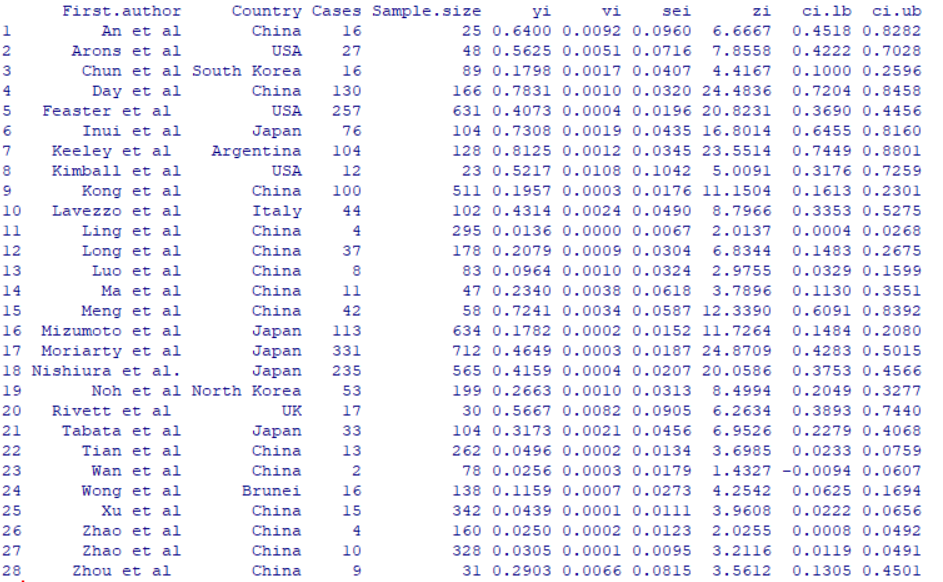

Supplement: S1 Table — (DOCX) [file pone.0249090.s002.docx]
