# Supplementary material for: Magnitude of asymptomatic COVID-19 cases throughout the course of infection: A systematic review and meta-analysis
Source: PLoS One. 2021 Mar 23;16(3):e0249090. doi: 10.1371/journal.pone.0249090 (PMC7987199; doi:10.1371/journal.pone.0249090)
Supplement: Attachment — Submitted filename: Response to Reviewers.docx [file pone.0249090.s003.docx]

**Author's response to reviews**

**Title**: **Magnitude of asymptomatic COVID-19 cases throughout the course of infection: a systematic review and meta-analysis**

**Author’s email addresses**

MA: [mulunehadis@gmail.com](mailto:mulunehadis@gmail.com)

LY: [lielt.yismaw@gmail.com](mailto:lielt.yismaw@gmail.com)

MAA: [agazhemoges@gmail.com](mailto:agazhemoges@gmail.com)

DBK: [danibekele2009@gmail.com](mailto:danibekele2009@gmail.com)

BM: [Belaynehmengist2008@gmail.com](mailto:Belaynehmengist2008@gmail.com)

BK: [bekalukassiedmu@gmail.com](mailto:bekalukassiedmu@gmail.com)

TYB: [yemanu.tilahun@gmail.com](mailto:yemanu.tilahun@gmail.com)

**Date: 12 December 2020**

**Dear Editor,**

We thank you for the chance to resubmit our revised version of the manuscript. Also, we would like to thank the reviewers for sharing their view and experience. The comments are very important that will improve the manuscript. The point-by-point responses for each of the comments are provided in the following pages. We hope that the revisions meet your standards and that the paper would be published in your journal. We look forward to working with you towards a final published product.

Sincerely,

Muluneh Alene, MPH

On behalf of co-authors

**Point by point responses to queries**

**Editor comments and suggestions**

This article aims to assess the magnitude of asymptomatic and pre-symptomatic COVID-19 cases which is essential and currently relevant for COVID-19 pandemic. Search terms may need to be refined to include as many as articles in the initial search. Will the authors consider to have pooled estimate stratified by age, setting such as hospital or community and study period to enrich the content. Also the exclusion criteria was not explicit mentioned. In terms of the language, authors are suggested to have substantial edit to arrive at the publishable quality.
**Response**

Thank you dear editor for your constructive comments and suggestions. In the revised version of the manuscript, we already refined the searching terms in order to include the existing potential studies. Unfortunately, the original studies were not report the magnitude of asymptomatic COVID-19 cases by age. Additionally, all studies included in this review are facility-based. We try to write the manuscript in acceptable quality of English language.

**Reviewer#1**

**Comments/suggestions #1**

Severe Acute Respiratory Syndrome Corn babirusa 2, instead of Sever respiratory syndrome Coronavirus 2

**Response#1**

Thank you dear reviewer for your constructive suggestion and comments. The comments are very important to improve the manuscript. We used the standard WHO name of the novel coronavirus (Sever respiratory syndrome Coronavirus 2).

**Comments/suggestions #2**

This systematic review and meta-analysis aims to determine the pooled magnitude of asymptomatic COVID-19 cases throughout the course of infection? What about total cases, pre-symptomatic cases? The course of infection or the course of outbreak?

**Response#2**

Thank you. The scope (objective) of this study was to determine the overall magnitude of asymptomatic COVID-19 cases throughout the course of infection using available evidences. In this review, we only consider patients who are asymptomatic (without sign and symptoms) in the course of infection, not outbreak.

**Comments/suggestions #3**

-Systematic review does not only include observational studies
- Study setting: worldwide? At hospital? At community?
- Population: All age group? Please provide age range.
- Publication status: I would not include unpublished articles
- Exclusion criteria: Not clear. Please explain.
- Outcome variable and data extraction: Please provide more details on the “predestined data extraction form”. The 1st section: How to assess the methodological quality of each study? The 2nd section: How to evaluate the comparability of the study group? The 3rd section: How to assess the outcome and statistical analysis?

**Response#3**

Thank you. We considered your comments and suggestions. We understand that systematic review and meta-analysis is not only for observational studies, but also it is applicable for experimental studies. However, in this study, we only consider observational articles. In addition, articles included in this review were facility-based. No restriction in age group. Furthermore, studies included in this systematic review were published and unpublished articles. Moreover, we revised the text in the section of exclusion criteria, outcome variable of the interest, and extraction form. Regarding, the quality assessment the following points are considered. 1) To assess the methodological quality of each study; we consider: representativeness of the sample, sample size, non-respondents, and ascertainment of the exposure (risk factor). 2) To evaluate the comparability of the study group; we consider: how studies control for the most important factor, and the study control for any additional factor.

3) To assess the outcome and statistical analysis; we consider: the statistical test used to analyze the data is clearly described and appropriate, and the measurement of the association is presented, including confidence intervals and the probability level.

**Comments/suggestions #4**

- What about incubation period and serial interval?
- Please explain how to perform sensitivity analysis

**Response#4**

Thank you. Incubation period is the time from infection occurred to the onset of signs and symptoms. Also, the serial interval is the time from illness onset in the primary case to illness onset in the secondary case. It also measured from pairs of cases with a clear infector–infectee relationship. In the revised version of the manuscript, we explained in detail how sensitivity analysis performed.

**Reviewer#2**

**Comments/suggestions#1**

Page-3: “Up to date, there are more than 22 million total cases with 780,000 deaths, worldwide.”, “Up to date” is not a right phrase to use here. Provide specific date of extracting data instead. Also, suggest to revise it as the study reporting the digits from long back in July, 2020.

**Response #1**

Dear reviewer, we thank you for your constructive comments and suggestions. The comments are very important to improve the manuscript. In the revised version of the manuscript, we provided the specific date with the current level COVID-19 cases and deaths.

**Comments/suggestions#2**

Page-3: “Studies vary depending on the number of study participants recruited, the type of design employed and the country in which the study conducted.” The sentence is not clear enough. What is the meaning of “the number of study participants recruited”? Need to revise the sentence for clarity. Further authors used the terms “Studies vary”, but not clearly mentioned in what context.

**Response#2**

Thank you. Yes, we learnt that the sentence needs revision, and we already revised it in the revised version of the manuscript. In other word “the number of study participants recruited” means the sample size for a particular study. In addition, we describe in detail that in what context study varies.

**Comments/suggestions#3**

Page-3: Continuation of point 2 above, author need to present the research potential and motivation of such review study in this paragraph before mentioning the objectives. Though author has mentioned the combining the exiting finding might strengthen the evidence.

**Response#3**

Thank you. We try to explain further the potential and motivation of this study, in the revised form of the manuscript.

**Comments/suggestions#4**

Page-4: “In addition, we searched from the reference lists of the included studies to identify any other studies that may have been missed by our search strategy.”, I fell such statement is redundant as it’s a part of the review when reviewers need to assess the main text along with the title and abstract.

**Response#4**

Thank you. We considered to revise it.

**Comments/suggestions#5**

Page-4: “Our search was performed between the 1st of June and the 15th of July, 2020.” the review time window is much backdated. Possibly suggest to update the time window to catch more data.

**Response#5**

Thank you. We already updated the searching time.

**Comments/suggestions#6**

Page-4: “Inclusion criteria”, the term ‘Design’ is not really referring the design of the reviewed studies, instead it’s the outcome variable for which the review is subject to. It preferably better of mentioning “Estimates/ Estimates reported” instead of “Design”? Else clarify.

**Response#6**

Based on the suggestion given, we mentioned “Estimates reported” instead of “Design”.

**Comments/suggestions#7**

Page-4: “Exclusion criteria” need to mention clearly. Current form is not conveying what author wanted to mention. Need more detailed criteria.

**Response#7**

In the revised version of the manuscript, we provided clearly the exclusion criteria of this study.

**Comments/suggestions#8**

Page-4: “Two experienced authors (MA and LY) extracted all essential data from the included studies using a predesigned data extraction form.” hope these two review author did the search independently. If yes, please mention it else no need to mention specifically in the text. Independent search one if the tools to ensure the reproducibility of the results.

**Response#8**

Thank you, we removed the name of the authors upon your suggestion.

**Comments/suggestions#9**

Page-5: “In addition, 10 articles were removed due to not extractable result and the outcome of interest not reported.” This 10 studies were excluded after assessing the related “Text” of the articles. Need to be clearly mention it, as mentioned similarly for “titles and abstracts”.

**Response#9**

In the revised manuscript, we explicitly mention the reason for excluding studies.

**Comments/suggestions#10**

Page-6: Table 1, “Study period” is not mentioned clearly for some of the studies. Need to clearly have a note for this in the table. Wonder, is it due to the missing information in the text.

**Response#10**

Thank you. Some original studies did not explicitly provided the study period (data collection period).

**Comments/suggestions#11**

Page-7: Author presented the magnitudes of ‘asymptomatic’ COVID-19 cases, wonder if it is included the ‘pre-symptomatic’ cases as well. Need clarification. Accordingly, the title of the manuscript should be revised.

**Response#11**

Thank you. The outcome of interest (objective) of this study was to estimate the pooled magnitudes of asymptomatic COVID-19 cases throughout the course infection. We will come on that with another article.

**Comments/suggestions#12**

Page-9: “We also compared the proportion of asymptomatic SARS-CoV-2 infections with previously emerged coronavirus pandemics.” I wonder whether the MERS was a Pandemic. Otherwise, better to be specific by using ‘pandemics/epidemics’ or just simply ‘outbreaks”.

**Response#12**

Thank you. We restated it.

**Comments/suggestions#13**

Page-9-10: “The result of this study suggest that contact and symptom based screening might fail to identify all potential SARS-CoV-2 infections.” Author clamed this without any discussion in the main text on it. I would prefer to illustrate this in the discussion section first with the results and then suggest in the conclusion. Conclusion should be related to the results, otherwise take-home massage will be weaker to the readers.

**Response#13**

Thank you. We already revised it.

**Comments/suggestions#14**

Page-9-10: “Scale-up mass testing, which targeting high risk populations is recommended to tackle the pandemic. Continuation of the above points, ‘mass testing’ and ‘testing of the target high risk group’ are two different things. Prefer to revise or rearrange the sentence here.

**Response#14**

Thank you. We already revised it.

**Comments/suggestions#15**

Many of the references were in preprint now published should be updated.
16. The references are not according to the PloS One specification (“Vancouver” style). Need to be updated.

**Response#15**

Thank you. We already updated the references according to the PLOS ONE requirement.

**Comments/suggestions#16**

Finally, I would suggest to the authors to give some effort in the presentation and language of the manuscript to avoid the redundancy of text and improve the language for general readers.

**Response#16**

Thank you dear reviewer. We try to write the manuscript in acceptable quality of English language.

**Thank you!!!**
